# Directionality of large-scale resting-state brain networks during eyes open and eyes closed conditions

**DOI:** 10.3389/fnhum.2015.00081

**Published:** 2015-02-19

**Authors:** Delong Zhang, Bishan Liang, Xia Wu, Zengjian Wang, Pengfei Xu, Song Chang, Bo Liu, Ming Liu, Ruiwang Huang

**Affiliations:** ^1^Department of Radiology, Guangdong Provincial Hospital of Chinese MedicineGuangzhou, China; ^2^Guangzhou University of Chinese Medicine Postdoctoral Mobile Research StationGuangzhou, China; ^3^Center for the Study of Applied Psychology, Key Laboratory of Mental Health and Cognitive Science of Guangdong Province, School of Psychology, South China Normal UniversityGuangzhou, China; ^4^School of Information Science and Technology, Beijing Normal UniversityBeijing, China; ^5^Institute of Affective and Social Neuroscience, Shenzhen UniversityShenzhen, China; ^6^Neuroimaging Center, University Medical Center Groningen, University of GroningenGroningen, Netherlands; ^7^National Key Laboratory of Cognitive Neuroscience and Learning, Beijing Normal UniversityBeijing, China

**Keywords:** resting-state fMRI, independent component analysis (ICA), Gaussian Bayesian network (BN), support vector machine (SVM), eyes closed, eyes open

## Abstract

The present study examined directional connections in the brain among resting-state networks (RSNs) when the participant had their eyes open (EO) or had their eyes closed (EC). The resting-state fMRI data were collected from 20 healthy participants (9 males, 20.17 ± 2.74 years) under the EO and EC states. Independent component analysis (ICA) was applied to identify the separated RSNs (i.e., the primary/high-level visual, primary sensory-motor, ventral motor, salience/dorsal attention, and anterior/posterior default-mode networks), and the Gaussian Bayesian network (BN) learning approach was then used to explore the conditional dependencies among these RSNs. The network-to-network directional connections related to EO and EC were depicted, and a support vector machine (SVM) was further employed to identify the directional connection patterns that could effectively discriminate between the two states. The results indicated that the connections among RSNs are directionally connected within a BN during the EO and EC states. The directional connections from the salience network (SN) to the anterior/posterior default-mode networks and the high-level to primary-level visual network were the obvious characteristics of both the EO and EC resting-state BNs. Of the directional connections in BN, the directional connections of the salience and dorsal attention network (DAN) were observed to be discriminative between the EO and EC states. In particular, we noted that the properties of the salience and DANs were in opposite directions. Overall, the present study described the directional connections of RSNs using a BN learning approach during the EO and EC states, and the results suggested that the directionality of the attention systems (i.e., mainly for the salience and the DAN) in resting state might have important roles in switching between the EO and EC conditions.

## Introduction

The human brain is a self-organized system in which multiple sub-systems complexly interconnect within a network (Bullmore and Sporns, 2009; Park and Friston, 2013). Many studies have demonstrated that eye behavioral states, such as when subjects have their eyes open (EO) or eyes closed (EC), could effectively modulate the spontaneous activity in various subsystems, e.g., the visual (Yang et al., 2007), sensorimotor (Marx et al., 2004; Liang et al., 2014), and default-mode network (DMN; Marx et al., 2004; Yang et al., 2007; Yan et al., 2009; Liang et al., 2014), the intersystem functional connectivity (Yan et al., 2009; Jao et al., 2013), and even the space organization of the interaction of these subsystems (Jao et al., 2013; Xu et al., 2014). However, the directional connections among these sub-systems within a network and their relation to eye behavioral states are not well understood.

Neuroimaging studies have demonstrated that spontaneous brain activity of functional subsystems overlaps highly with task-induced activity (Fox et al., 2006; Mantini et al., 2007; Zuo et al., 2010). These inherent spontaneous activities were intra-dependently organized within the subsystem but also functionally cooperated and communicated interdependently (Tononi et al., 1994; Sporns, 2013). In particular, the directional connectivity among these subsystems has been shown to be an important characteristic of the dynamics of the spontaneous activity (Friston et al., 2003; Liao et al., 2011; Liu and Duyn, 2013). The directional connections reflect the dependence relationships among subsystems when passing messages through the system. In fact, several models (e.g., the hierarchical brain systems model (Carhart-Harris and Friston, 2010) and the triple-network model (Menon, 2011)) have been proposed to depict the relationship among these subsystems (e.g., DMN, attention system, and executive-control systems). All these models focused on the directional interaction of these sub-systems during information transfer.

Recently, multi-variate pattern analysis (MVPA) approach has been widely applied to explore the linkage between the distributed functional signal and the brain states in fMRI studies (Craddock et al., 2009; Poldrack et al., 2009; Dosenbach et al., 2010; Shen et al., 2010; Naselaris et al., 2011; Zeng et al., 2012; Vergun et al., 2013). By using the MVPA approach, the category of the EO and EC conditions could be decoded from the spatially distributed neural activities patterns (Liang et al., 2014). In the present study, we hypothesized that the directional connections of brain subsystems could be discriminative between the EC and EO conditions. To this end, the independent component analysis (ICA) approach was used to identify the independent components (ICs) corresponding to the separate subsystems. Then, the directional connections among these subsystems related to EO and EC were measured by using the Gaussian Bayesian network (BN) learning approach. In order to investigate the difference of the directional connections between the two conditions, a MVPA approach, the recursive feature elimination (RFE)-based support vector machine (SVM) was further applied to identify the directional connection patterns that could effectively discriminate between the EO and EC states.

## Materials and methods

### Participants

In the present study, a total of 20 right-handed, healthy undergraduates/postgraduates (9 males/11 females, 20.45 ± 2.76 years) were recruited from the campus of Beijing Normal University. This study was approved by the Institutional Review Board of the Beijing Normal University Imaging Center for Brain Research. Written informed consent was given by each participant before the experiment.

### Data acquisition

The resting-state functional magnetic resonance imaging (rsfMRI) data were acquired on a 3T Siemens Trio TIM MR scanner equipped with a 12-channel phased array receiver-only head coil at the Imaging Center for Brain Research, Beijing Normal University. The rsfMRI data were obtained using a gradient-echo EPI sequence with the following two parameter sets: (A) TR = 2000 ms, TE = 30 ms, 33 transverse slices, slice thickness = 3.5 mm, gap = 0.7 mm, flip angle = 90°, FOV = 224 mm × 224 mm, matrix = 64 × 64, and 240 volumes covering the whole brain; (B) TR = 3000 ms, 40 transverse slices, slice thickness = 3.5 mm, no gap, 160 volumes, and the other parameters were identical to those of (A). For each parameter set, all participants were scanned under EC and EO conditions in turn. A total of 4 types of rsfMRI scans (i.e., AO, AC, BO, and BC) were obtained for each participant in the same session. In order to reduce the sequence effect, the order of data acquisition was counterbalanced across all participants. In addition, a high-resolution 3D brain structural image was also acquired for each participant using the MP-RAGE sequence with the implementation of the parallel imaging scheme GeneRalized Autocalibrating Partially Parallel Acquisitions (GRAPPA; Griswold et al., 2002) and an acceleration factor of 2.

### Data preprocessing

Only the rsfMRI data corresponding to TR = 2000 ms was analyzed for the present study. Data preprocessing was performed using DPARSF^1^ based on REST^2^ and SPM8.^3^ The first 10 volumes of the functional images were discarded to account for signal equilibrium and the participant’s adaptation to the scanning environment. We then corrected the remaining functional images for within-scan acquisition time differences between slices and realigned all images to the first volume to correct for head motion. This realignment procedure provided us with a record of head motion during each rsfMRI scan. None of the participants was excluded for excessive head motion based on criteria of >1 mm displacement or an angular rotation of >1° in any direction. The summary scalars of both gross (maximum and root mean square) and micro (mean frame-wise displacement) head motion were matched between the two conditions (all *p* > 0.14). The corrected functional images were subsequently spatially normalized to the MNI standard template using an optimum 12-parameter affine transformation and non-linear deformations and were re-sampled to a voxel size of 3 × 3 × 3 mm^3^. The resulting MRI data were smoothed by a Gaussian filter with a full width at half maximum of 4 mm and were further temporally band-pass filtered (0.01–0.08 Hz) to reduce the effects of low-frequency drift and high-frequency physiological noise.

### Group ICA

The resting-state networks (RSNs) of the brain were identified by using ICA analysis, which were further used for the subsequent RSNs interconnectivity analysis via the BN approach. Here, the Group ICA program was completed using the fMRI Toolbox GIFT.^4^ The preprocessed data after band filter of all participants in the two conditions were first analyzed using principle component analysis (PCA) to reduce the data dimension. Here, two-step PCA was used: the data for each individual participant was temporally dimension-reduced, and then the dimensions were again reduced to the optimal numbers after concatenation across subjects within groups. The optimal number of ICs was estimated at 35 based on the minimum description length (MDL). Then, the data were separated by ICA using the Extended Infomax algorithm (Lee et al., 1999). After ICA separation, the mean ICs and the corresponding mean time courses over all the participants were used for the back-reconstruction of the ICs and the time courses for each individual participant (Calhoun et al., 2001). Then, the intensity values in each IC spatial map were converted to Z-scores, and a one sample *t*-test (False Discovery Rate, FDR, *p* = 0.01) was then performed under two conditions to determine the RSNs. The present study identified in total of 9 RSNs (Table 1) which would be used as the nodes of the BN models. Notably, these nodes were depicted by using a sphere (radius = 6 mm) in the peak of each RSN *t* map. There were two main considerations for selecting these RSNs. First, all of them have already been documented to be modulated by switching between EC and EO conditions (Yang et al., 2007; Yan et al., 2009; Jin et al., 2013; Xu et al., 2014; Yuan et al., 2014). Second, they are spatially distributed across different systems (i.e., default mode, attention, and unimodal sensory systems). Previous studies (Carhart-Harris and Friston, 2010) have suggested that these brain systems exhibit an obvious hierarchical organization attribute which is the physiological principle of brain region interaction. Thus, we speculated that directional connections among these identified RSNs could effectively reflect the modulation effect of brain states (EC or EO) on brain spontaneous activity.

**Table 1:** **Details of the selected RSNs**.

Index	RSNs	Regions	Coordinates			***t***-value	***r***-value
			*X*	*Y*	*Z*		
1	PVN	Lingual_R	6	−84	−3	18.75	0.52*
2	HVN	Occipital_Sup_R	24	−102	9	19.24	0.34*
3	PSMN	Supp_Motor_Area_R	3	−15	69	17.98	0.37*
4	VMN	Postcentral_R	60	−6	33	18.88	0.50*
5	DAN	Parietal_Sup_L	−18	−75	51	19.68	–
6	CEN	Frontal_Inf_Tri_R	51	27	27	14.33	0.46*
7	aDMN	Frontal_Sup_Medial_L	0	54	15	18.59	0.55*
8	pDMN	Cingulum_Mid_R	3	−30	30	25.31	0.64*
9	SN	Cingulum_Ant_L	−9	39	−3	24.27	0.50*

Note: The RSNs are the same as those in Figure 2. The coordinates are the peak voxel value of each RSN in MNI space. The regions are those in which the peak voxels were located. The brain regions are reported according to the Anatomical Automatic Labeling (AAL) template. The *t* value indicates the scores of the one-sample *t*-test on the individual IC pattern (*p* < 0.001, FDR corrected). The *r* value indicates the scores of the spatial similarity index between the individual IC pattern and resting-state fMRI templates derived from previous studies^5^, *p < 0.05.

**Figure 1:**
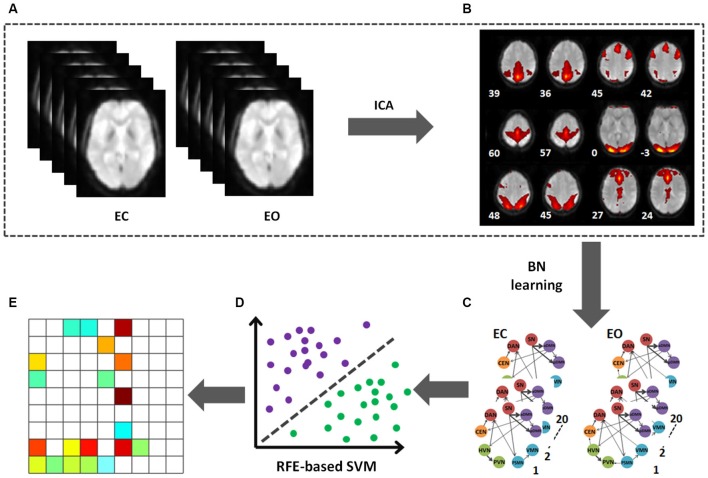
**Flowchart of data processing. (A)** The preprocessed data after band filter of two conditions (EC and EO). **(B)** The mean ICs. **(C)** BN connectivity patterns on the selected 9 ICs in EO and EC. **(D)** The RFE-based SVC was used to identify the discriminative pattern. **(E)** The pattern able to effectively discriminate between the EO and EC states.

**Figure 2:**
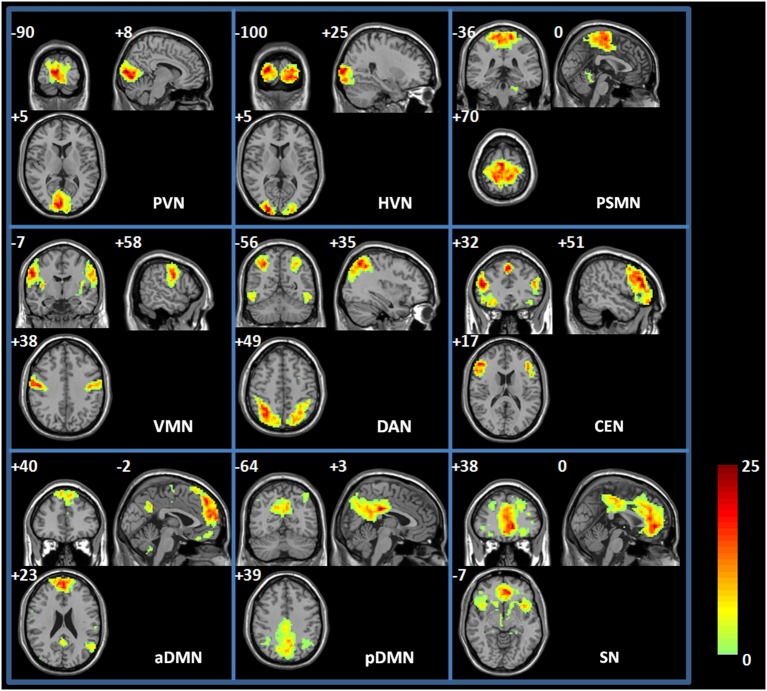
**Spatial map of each RSN**. PVN, primary visual network; HVN, high-level visual network; PSMN, primary sensory-motor network; VMN, ventral motor network; SN, salience network; DAN, dorsal attention network; CEN, central executive network; aDMN; anterior default-mode network; and pDMN, posterior default-mode network. Each RSN map was the result of a one-sample *t*-test on the individual IC pattern (*p* < 0.001, FDR corrected).

### Gaussian BN method

The directional connections among the RSNs within a large-scale BN were measured related to EO or EC conditions. As we know, BN is a data-driven statistical technique that can measure the condition dependencies among nodes within a network. The dependencies are qualified by the conditional probability of each node given its parent nodes in the network. In fact, the BN learning approach has already been widely applied in the fMRI studies (Kim et al., 2008; Li et al., 2008, 2011; Wu et al., 2011).

In the present study, the time series of the RSNs were extracted for the BN analysis, to this end, a linear regression was performed to remove the effects of 9 nuisance covariates (i.e., the global mean signal, the white matter signal, the cerebrospinal fluid signal, and 6 head motion parameters) of the preprocessed fMRI data which were used the RSN identification. Then, the resting-state time series of each node were extracted by averaging the intensities over all voxels within the sphere (radius = 6 mm) at each time point for each individual under EO and EC conditions. Finally, the Gaussian BN method was applied, and the identified ICs corresponded to the node in the BN; their time series was assumed to be Gaussian distributed. A Bayesian information criterion (BIC)-based (Schwarz, 1978) BN learning approach was adopted to identify the optimal BN. In this process, the optimized BIC score among the space of possible candidate networks was selected. The structure and parameters of the BN were evaluated using the L1-Regularization Paths algorithm (Schwarz, 1978) and maximum likelihood (ML) estimate implemented by the collection of Matlab functions called L1 DAGLearn^6^ and Bayesian Net Toolbox (BNT).^7^ Herein, several radius sizes (i.e., 3 mm, 6 mm, and 12 mm) were applied to define each node sphere for extracting time series in BN analyses, to evaluate the radius-size effect on the final BN properties.

### Machine learning method

The LIBSVM toolbox^8^ with the linear support vector classification (SVC) was applied as the classifier for the MVPA (Zhang et al., 2013). In the present study, the RFE-based SVC was used to identify the directional connection pattern that could effectively discriminate between the EO and EC states. The directional connectivity in the BN (*n* = 81, i.e., 9 RSNs × 9 RSNs) were extracted to be as the discriminative feature. The RFE approach was first applied to rank these features according to their ability to discriminate between EO and EC. Then, we recursively selected the most important features (e.g., the first one, the first two, the first three, and so on) to constitute the discriminative patterns. The performance of these constituted patterns in state (EC and EO) classification was further investigated using the SVC classifier. Here, the leave-one-out approach was used to validate these patterns’ performance in state classification. More detailed information is described in a previous study by our group (Zhang et al., 2013). The whole framework for the present study is shown in Figure 1.

## Results

### RSN maps for EC and EO states

The group ICA method was used to identify the RSNs of the spontaneous activity of the resting state during the EO and EC states. Figure 2 shows the spatial maps of the RSNs derived from the two states from 20 young participants. RSNs from the 35 ICs were selected for the present study, and these RSNs were spatially distributed across the cerebral cortex and maximally overlapped with the previously reported primary visual network (PVN) (e.g., the bilateral calcarine regions and lingual regions), high-level visual network (HVN) (e.g., the bilateral supperior occipital regions and middle occipital regions), primary sensory-motor network (PSMN) (e.g., the bilateral paracentral lobule (PCL)), ventral motor network (VMN) (e.g., the bilateral postcentral regions), salience network (SN) (e.g., the bilateral anterior cingulum regions, medial frontal regions, and insula regions), dorsal attention network (DAN) (e.g., the bilateral superior parietal regions and inferior temporal regions), central executive network (CEN) (e.g., the bilateral superior medial frontal regions, inferior frontal regions, and supplementary motor area), anterior default-mode networks (aDMN) (e.g., the left angular region, the bilateral superior medial frontal regions and precuneus regions), and posterior default-mode networks (pDMN) (e.g., the bilateral angular regions, post cingulum cortex, and precuneus regions). Table 1 shows the detailed information of each RSN. These identified RSNs are highly consistent with those previously reported (Damoiseaux et al., 2006; Mantini et al., 2007; Sridharan et al., 2008; Jann et al., 2010; Li et al., 2011).

### Directional connectivity of RSNs

**Figure 3:**
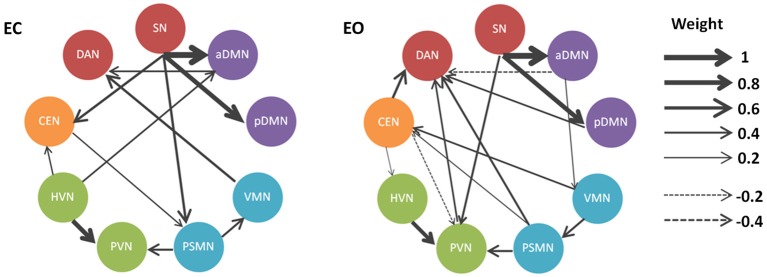
**Directional connectivity patterns related to the EO and EC states in the BN model**. The RSNs (sphere radius = 6 mm) are graphically connected to depict their conditional dependencies in a BN model. Only connections that survived the significance testing (*p* < 0.05) are shown. Solid and dashed arcs correspond to the positive and negative connections, respectively. Line width is proportional to the connection weights.

After identifying the RSNs using the Group ICA method, we further applied the BN approach to explore the directional connectivity among these RSNs. We found that there was directional connectivity among these RSNs during the EO and EC states. Figure 3 shows the BN-based connectivity pattern among these RSNs (sphere radius = 6 mm) in the BN. Detailed information is listed in Table 2. The BNs related to the three radius sizes are highly consistent.

**Table 2:** **Directional connectivity and its weight in BNs related to EC and EO**.

Direct Connections	Weight coefficients	
	EC	EO
SN→ADMN	1.42	1.59
SN→PDMN	0.85	0.87
HVN→PVN	0.8	0.67
PSMN→PVN	0.37	0.34
ADMN→DAN	0.32	−0.21
VMN→DAN	0.43	–
SN→CEN	0.41	–
SN→PSMN	0.35	–
HVN→ADMN	0.28	–
CEN→DAN	–	0.44
SN→PVN	–	0.36
PSMN→DAN	–	0.33
VMN→CEN	–	0.31
PDMN→DAN	–	0.30
PVN→DAN	–	0.29
ADMN→VMN	–	0.16
CEN→PVN	–	−0.16
PSMN→VMN	0.33	–
VMN→PSMN	–	0.35
CEN→PSMN	0.24	–
PSMN→CEN	–	0.19
HVN→CEN	0.21	–
CEN→HVN	–	0.14

*Note: All the listed connections survived significance testing (*p* < 0.05) during the stepwise regression analysis within each condition*.

Our results demonstrated that the directional connectivity from the SN to the DMN (i.e., aDMN and pDMN) and from the HVN to the PVN were salient in the resting-state BN based on the directional connectivity weights in the BN. In particular, the most weighted directional connectivity was observed in the SN outgoing connections to aDMN and to pDMN, as well as HVN outgoing connections to PVN. Moreover, we also noted that the directional connectivity mentioned above was consistent between the EO and EC states (Figure 3). Of course, the consistent directional connectivity between states was observed not only in those with high weights but also in connections from PSMN to PVN.

In addition to the robust directional connectivity with high weights in the BN network, the dynamic and directional properties of several RSNs were also consistent. We found that the SN and DAN were unique among these RSNs. The SN and DAN perform oppositely in the BN network: the former mainly sent out connections to other networks, while the latter mainly received connections from other networks.

There were significantly discriminative directional connectivity patterns in the BN networks related to EO and EC. We found that several specific connections, including SN to CEN, VMN to DAN, SN to PSMN, and HVN to aDMN, were observed in the BN network related to EC. The connections such as CEN to DAN, pDMN to DAN, PVN to DAN, CEN to PVN, PSMN to DAN, VMN to CEN, aDMN to VMN, and SN to PVN were found in the EO state. In addition, we noted that EO and EC changed the directional connectivity between RSNs. For instance, the connections between PSMN and VMN, CEN and PSMN, and CEN and HVN were all existing connections in the BN network, but the direction was reversed between the EO an EC states. In particular, we found that the connection pattern of the PVN was quite different in the BN network related to EO compared to the network related to EC. The directional connections in the EO state were increased when compared with those of the EC state (Figure 3).

### SVM classifier performance

**Figure 4:**
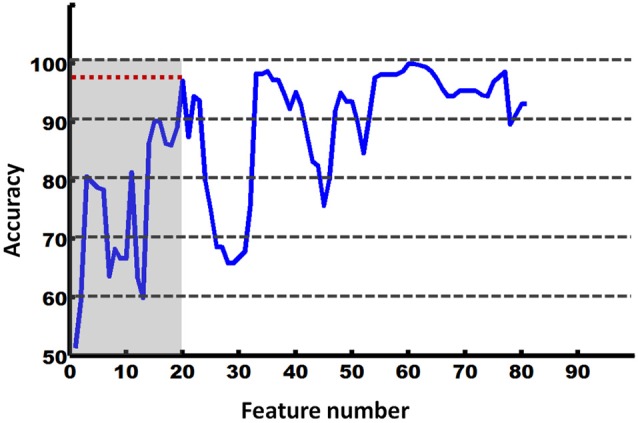
**Accuracy of RFE-based SVM classification between the EO and EC states**.

The RFE-based SVC was used to further explore the directional connectivity patterns that could effectively discriminate between the EO and EC states. We found that the first 20 directional connections (according to the RFE results) form a pattern that could effectively discriminate between these two states (accuracy = 97%). The details are shown in Figure 4.

**Figure 5:**
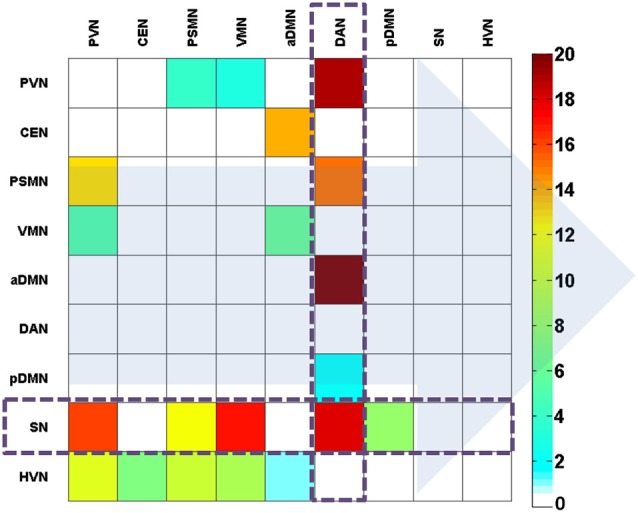
**The effectively discriminative pattern of the EO and EC states based on the weight coefficients of the direct connections**. The background arrows represent the direction of connections. The color bar represents the contribution in the discrimination between the states. The color describes the level of contribution in classification.

We further investigated these features (directional connectivity) in the created discriminative pattern. This pattern is shown in Figure 5. The results indicated that the salient feature that helped classify the two states was the attention (i.e., SN and DAN)-related directional connectivity in the BN. Notably, the sub-systems of the attention network performed oppositely. The DAN primarily received the connections from the other networks, whereas the SN primarily sent out connections to other networks, and this was the most salient feature of the discriminative pattern (Figure 5).

## Discussion

The present study investigated the directional connections among the large-scale RSNs in the human brain related to having EO and having EC by combining ICA and BN learning approaches. The main findings can be summarized as follows: (i) the directional connections from SN to DMN (aDMN and pDMN) and from HVN to PVN were obvious characteristics of the BN network (both EC and EO states); and (ii) the identified discriminative pattern was primarily characterized as the attention (i.e., SN and DAN)-related directional connections in the BN network, which performed well in EO and EC classification, with the SN and DAN exhibiting opposite dynamic and directional properties in the EO and EC states.

### Resting-state BN

Many previous studies have demonstrated that the SN, DMN, and CEN were core neurocognitive networks of the human brain (Greicius et al., 2003; Fox and Raichle, 2007; Seeley et al., 2007). Specifically, SN is involved in the capture of biologically and cognitively relevant events (Seeley et al., 2007; Sridharan et al., 2008; Menon and Uddin, 2010), DMN plays important roles in monitoring the internal mental landscape (Greicius et al., 2003, 2004; Qin and Northoff, 2011), and CEN is crucial for a range of cognitively demanding tasks (e.g., working memory, problem solving, and decision making) (Miller and Cohen, 2001; Koechlin and Summerfield, 2007). According to the triple network model (Menon, 2011), the interactive connections among these three networks are highly involved into the cognitive and affective functions of the human brain, and aberrant connections play a prominent role in brain dysfunction in several disorders.

In the present study, we found that the SN and the DMN were hub regions of the resting-state BN. Moreover, our results showed that there was significant outflow of signals from the SN to other systems. In particular, the directional connections from the SN to the anterior/posterior DMN were the obvious features of the resting-state BN. In addition, this feature was highly consistent between the EO and EC states (Figure 3). In fact, the directionality among these three systems (i.e., SN, DMN, and CEN) has been measured in a previous study (Sridharan et al., 2008) using chronometric techniques and Granger Causality Analysis (GCA) in three experimental conditions. The findings of this previous study indicated that the SN (e.g., right fronto-insular cortex) plays a critical and causal role in switching between the CEN and the DMN. Obviously, all these findings correspond to the notion that the SN was important in detecting and mapping salient external inputs and internal brain events. That is, the SN is an integral hub (Menon, 2011) in mediating dynamic interactions between other large-scale brain networks involved in externally oriented attention and internally oriented self-related mental processes.

In addition, our results suggested that the outflow from the HVN to the PVN was also a salient feature of the resting-state BN, which was also highly consistent between the EO and EC states. Several previous studies have investigated the spontaneous activity of these visually associated regions. The collective findings suggest that visual-related spontaneous activity may be highly associated with mental operation processing such as memory-related mental imagery and visual memory consolidation processes (Wang et al., 2008). In addition to the directional connection from HVN to PVN, we also found a directional connection from the PSMN to PVN. In particular, CEN-related directional connections were observed in the resting-state BN. However, we noted that the directionality of these connections was reversed between EC and EO states. Few studies have examined the role of the CEN in the EC and EO switch. This restricted our further speculation on the findings regarding the CEN that need to be studied in depth in the future. Considering the high involvement of the CEN in working memory related mental operations (Babiloni et al., 2004; Kondo et al., 2004), our findings may also suggest that the visual memory-related mental operations are predominant in the spontaneous activity of the human brain.

### Directional connection change between EC and EO

Recent findings have demonstrated that there are distinct mental states related to the EO and EC states. Specifically, there is an “exteroceptive” mental activity state characterized by attention and ocular motor activity during EO and an “interoceptive” mental activity state characterized by imagination and multisensory activity during EC (Marx et al., 2003, 2004). Accordingly, many studies have shown that eye behavioral states (e.g., EO and EC) modulated the brain spontaneous oscillation states within several systems, such as the visual system (Yang et al., 2007) and DMN (Yan et al., 2009). The primary contribution of the present study was to further investigate the directional connections among these systems in the resting-state brain during the EO and EC states and explore the differences in the directional connection between these systems during different states. In particular, we applied a RFE-based SVM approach to identify a discriminative pattern that performed well in EO and EC classification. After analysis of this pattern, we found that the dynamic and directional properties of the SN and DAN were opposite: SN gave out information to other systems, and the DAN received information from other systems (Figure 5). These findings were consistent with the notion of Posner et al. that the DAN and SN possessed different roles in brain function, i.e., the DAN is primarily in charge of prioritizing sensory input, and the SN are highly related to top-down task control (Petersen and Posner, 2012).

Accumulated evidence suggests that the “salience system” and the “dorsal attention system” are the two main subsystems of the attention system (Seeley et al., 2007). We also found that these two subsystems played different roles in pattern discriminating between the EO and EC states. Considering that the SN and DAN are two subsystems of the attention system and previous studies have shown that the SN is crucial as an integral outflow hub for initiating network switching, the attention network may have an important role in switching between the EO and EC states. The subsystems of the attention system may be coordinated in the dynamic translation of information in the resting-state BN. More details on the role of the attention system in the resting-state BN should be further explored. Nevertheless, our findings provide new evidence for the important role of the attention-related directional connections in the discrimination between the EC and EO states.

Many studies have tested the roles of the attention system in switching of mental states (Wylie et al., 2003; Elton and Gao, 2014). However, few studies investigated the neural properties of attention network during resting-state. As most of the knowledge about the attention network functions are derived from the task related studies (Hampton and O’Doherty, 2007; Schafer and Moore, 2007; Sridharan et al., 2008), which limited our interpretations of this study. Although the relationship between the RSN and task neural activity has been reported in many studies (Hermundstad et al., 2013; Cole et al., 2014), the exact relation between them is still unclear. In the present study, we found that the DAN-related directional connections were changed between the EC and EO conditions; especially, we found that more information from primary sensory modalities (e.g., PVN, PSMN) was input into the DAN in the EO condition when compared to the EC condition. This observation was consistent with several previous studies (Marx et al., 2003, 2004; Brandt, 2006) which proposed the activated attention system in the “exteroceptive” network whereby eyes-open periods (McAvoy et al., 2012).

The PVN-related directional connection was also different between the EO and EC states. Currently, there are still controversial viewpoints about the neural activity change in the visual cortex between the EO and EC states. For example, the lower amplitude of low-frequency fluctuation (ALFF) in the bilateral visual cortex and higher ALFF in the right PCL were observed during EC compared with EO (Yang et al., 2007). The possible interpretation of this result was that the visual cortex was activated by visual input during EO (Raichle et al., 2001; Uludağ et al., 2004). However, several studies contrarily indicated that EC activated the neural activity of visual areas (Marx et al., 2003, 2004). The present study provided new evidence for the PVN-related neural activity pattern change between the EC and EO states. We found that more directional connections were shown to be related to the PVN during EO compared with EC. In particular, a salient directed connection was observed from PVN to DAN during the EO condition. This finding also confirmed the hypothesis proposed by Marx et al., that the EO condition corresponds to the “exteroceptive” network.

### Limitations

There are several issues that should be addressed in future works. First, the present study investigated the directional connection among RSNs within a BN. The RSNs related to EO and EC were the focus, but not all the RSNs were included. Therefore, the directional connections of other RSNs should also be detected using a similar approach. Second, the directional connections of the spontaneous activity among large-scale systems were explored in a low-frequency band (0.01–0.08 Hz) in the present study, and the brain activity differences between EC and EO were also observed in a high-frequency band (Jin et al., 2014; Yuan et al., 2014). It may be interesting to explore the directional connections of brain systems in other frequency bands. Third, the present study investigated the changes of dynamical connections of RSNs between EC and EO conditions, the main findings should be further expanded among other conditions (e.g., EC, EO, fixed condition; Yan et al., 2009; Patriat et al., 2013). Forth, a limited number of RSNs were used to preliminarily explore the directionality of resting-state brain networks during EC and EO conditions, other RSNs (e.g., the audition network and emotion network) should be considered in future studies. Finally, there may be confusion factors (e.g., eye movements) in this study, further studies are necessary to reproduce our main findings using new methods with the development of this field.

## Conclusion

Collectively, the present study investigated the dynamics and directionality of the large-scale RSNs within a BN during the EO and EC states. We found that the salient features of the resting-state BN (EC and EO) were the directional connections from SN to DMN and from HVN to PVN. However, the differences in the BN between EC and EO were observed in the attention (SN and DAN)-related directional connections in the BN network. These results demonstrated the dynamics and directionality of the attention systems within a BN that were important in switching between the EC and EO states.

## Conflict of interest statement

The authors declare that the research was conducted in the absence of any commercial or financial relationships that could be construed as a potential conflict of interest.

## Footnotes

^1^http://www.restfmri.net/forum/DPARSF

^2^http://restfmri.net

^3^http://www.fil.ion.ucl.ac.uk/spm/

^4^http://icatb.sourceforge.net/

^5^http://www.brainnexus.com/resources/resting-state-fmri-templates

^6^http://www.cs.ubc.ca/~murphyk/Software/DAGlearn/

^7^http://code.google.com/p/bnt/

^8^http://www.csie.ntu.edu.tw/~cjlin/libsvm/
